# Development and application of fluorine doped bismuth vanadate reduced graphene oxide Nafion composite electrode as an electrochemical sensor for 4-chlorophenol

**DOI:** 10.1038/s41598-023-49205-y

**Published:** 2023-12-11

**Authors:** Bahareh Ghorbannejad, Alireza Mahjoub, Nima Dalir

**Affiliations:** 1 Department of Inorganic Chemistry, Faculty of Basic Sciences, Tarbiat Modares University, P.O. Box 14115-175, Tehran, Iran; 2 Department of Renewable Energy, Faculty of Interdisciplinary Sciences and Technologies, Tarbiat Modares University, P.O. Box 14115-175, Tehran, Iran

**Keywords:** Environmental sciences, Chemistry, Materials science

## Abstract

This study describes the synthesis of fluorine-doped bismuth vanadate (F_0.1_BiVO_4_) and its composite with graphene oxide (GO) to improve charge transport properties. Based on the structural and morphological analysis such as X-Ray diffraction (XRD), Fourier Transform Infrared Spectroscopy (FT-IR), Scanning Electron Microscopy (SEM), and RAMAN the composite of F_0.1_BiVO_4_/r-GO/Nafion was successfully prepared with no filth. It was used to selectively detect the environmental contaminant 4-chlorophenol (4-CP) on a modified glassy carbon electrode (GCE). The electron channeling ability of reduced graphene oxide (r-GO) with F_0.1_BiVO_4_ yielded a great electrochemical response (ER) in cyclic voltammetry compared to pure GCE and other modified electrodes. The differential pulse voltammetry response of 4-CP was highly sensitive with the detection of limit (LOD) of 0.56 nM and a wide linear response of 0.77–45.0 nM. Fluorine doping, in particular, was able to affect the crystal growth of BiVO_4_, which was the primary cause of the aforementioned improvement. On the other hand, r-GO acts as an electron bridge to improve charge transfer between electrolytes and F-BiVO_4_ due to its high electron transport rate. These results demonstrate the effectiveness of F_0.1_BiVO_4_/r-GO/Nafion/GCE for the electrochemical detection of 4-CP.

## Introduction

Globally, water pollution poses a threat to human health and is receiving increasing attention. Organic compounds like chlorophenols have been ranked among the most hazardous organic pollutants due to their widespread use in agriculture and industry^1^. They are widely used as wood preservatives, industrial adhesives, disinfectants, pesticides, paints (in cans), and antiseptics in industrial applications as well as paper and plastics manufacturing and plant growth regulators, and intermediates in the production of pharmaceuticals^2–4^. Among chlorophenol derivatives, 4-chlorophenol (4-CP) is the most toxic and hazardous^1^. The high toxicity, mutagenic, and carcinogenic effects of 4-chlorophenol (4-CP) on the ecosystem and human health, as well as its entry into the food chain, have caused increased public concern in recent years^2^. Therefore, there is a need for a reliable, rapid and sensitive method to analyze the environment and potable water, and also to prevent harm to human health. Up to now, several quantitative strategies have been developed for the quantification of 4-CP, such as UV spectrophotometry, enzyme-linked immunosorbent assay (ELISA), capillary electrophoresis, fluorescence spectroscopy, and high-performance liquid chromatography (HPLC)^5–7^. Nonetheless, the above-mentioned methods necessitate large and expensive equipment, skilled operators, and time-consuming processes that restrict the scope of applications. Among 4-CP detection procedures, methods based on the use of electrochemical sensors (ECSs) have enjoyed considerable attention because of their rapid response speed, high sensitivity, and low cost^8,9^. In recent years, developing ECSs for the detection of 4-CP through modified electrodes has enjoyed considerable attention. The main components in developing electrodes are noble metal nanoparticles (NPs) and enzymes^10,11^. Two drawbacks that lead to the low reproducibility of sensors are immobilization difficulty and instability, which are regarded as the main disadvantages of enzyme-modified sensors. Moreover, electrodes modified with noble metal NPs (e.g., gold, silver, and platinum) are too expensive. One of the most important methods for increasing the sensitivity of sensors is the surface modification of a nano-sized core, which is a heterostructure with two or more materials at a nanometer scale in a hybrid structure. The unique physical and chemical properties of hybrid structures make them encouraging candidates for applications in various fields like biomedicine and electronics^12^.

In recent years, various metal oxides have received significant attention as electronic mediators with high potential for the fabrication of effective chemical sensors^13–16^. For instance, Uddin et al.^14^ reported that ZnO/RuO_2_ nanoparticles exhibited excellent sensitivity toward 2-nitrophenol. In another study, Ge-dopped ZnO composite on flat GCE was fabricated by electrochemical method. It has provided a better response compared to simple flat GCE or other electrodes in detection of 4-aminophenol^13^. Several reports have also shown that bismuth vanadate (BiVO_4_) nanoparticle-based photoelectrochemical sensors exhibit a good response for the detection of nitrite anions (NO_2_^−^)^17^ and non-enzymatic H_2_O_2_ photoelectrochemical sensors^18,19^. In addition to having a narrow energy gap of 2.4 eV, it has other unique characteristics, including excellent physicochemical properties, easy fabrication, good conductivity, low cost, chemical stability in aqueous solutions and non-toxicity^11,12^. Despite having a high electrical conductivity, pure BiVO_4_ (P-BiVO_4_) tends to agglomerate and reduces the fast charge and discharge capabilities due to its small specific surface area^20^. To overcome these drawbacks, many researchers have applied graphene oxide as a substrate for BiVO_4_. The large surface area of electrode materials has been regarded as important for enhancing the sensing capability. The development of reduced graphene oxide (r-GO), has been recently carried out to avoid the irreversible agglomeration. Gr has unique properties^21,22^, which provide r-GO with advantages such as high electron transport rate, high mechanical strength, and large specific surface area^23,24^. These advantages make r-GO to be a promising sensing substrate, on which hydrophilic groups serve as an electron bridge for enhancing the charge transport between BiVO_4_ and electrolytes.

Up to now, several studies have confirmed that metal and non-metal doping can enhance electron transfer efficiency in BiVO_4_ structure. For example, Rohloff and coworkers exhibited that the photo-electrochemical performance of Mo:BiVO_4_ photoanodes achieved 21%^25^. Another study^3^ has revealed that Au doping into BiVO_4_ structure significantly improved water splitting performance. According to this study, Au plays a crucial role in visible light absorption and internal defects formation. Fluorine (F) doping in particular is capable of affecting the crystal growth of BiVO_4_, as well as providing an internal electric field that is helpful for separating photogenerated electron–hole pairs. Jiang and coworkers^26^ reported that Fluorine-dopped BiVO_4_ exhibited higher surface area, better light-absorbing performance, and as result of that lower bandgap energies, which enhanced photodegrdation of phenol to 97%.

**Figure 1:**
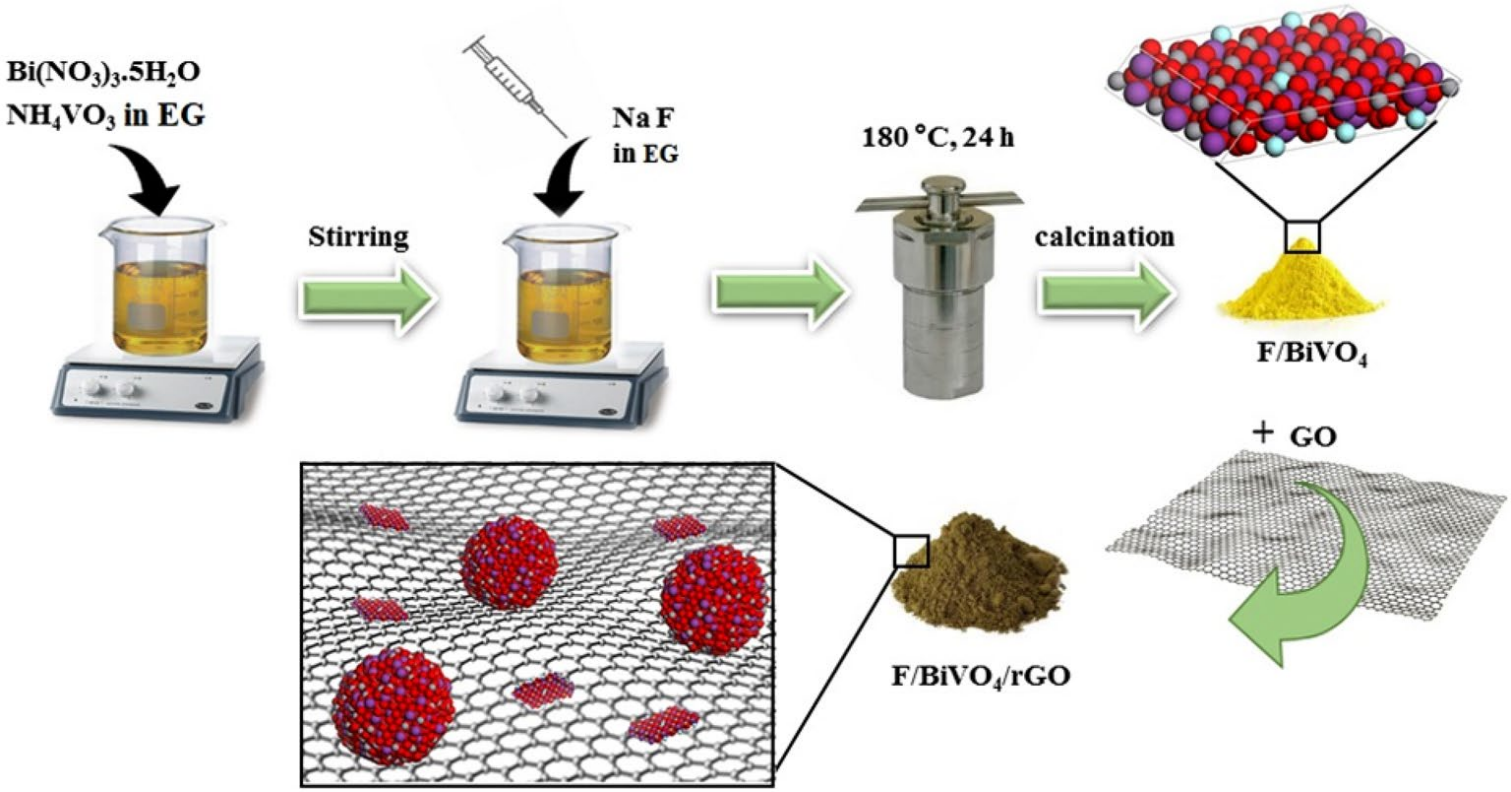
Synthesis method of F-doped BiVO_4_ and its composite.

In this work, a novel F_0.1_BiVO_4_/r-GO/Nafion composite was prepared using an effective three-step method (Fig. 1). First, F-BiVO_4_ was synthesized via a solvothermal method using ethylene glycol solvent under a cost-effective and mild reaction. This method enables the synthesis of materials with various crystal morphologies by adjusting synthesis parameters^27^. By incorporating F into BiVO_4_, defects are introduced into the lattice structure which leads to an increase in electron mobility and enhanced performance. Nonetheless, there is no report on the use of F as a template to synthesize BiVO_4_. Next, the r-GO, a 2D carbon nanosheet, was utilized as a substrate for immobilizing F-BiVO_4_. Then Nafion was applied to fabricated electrodes for stickiness (as chemical coating binder) of nanostructure F_0.1_BiVO_4_/r-GO onto GCE electrodes. One of the unique features of Nafion is its ability to facilitate proton transfer from its sulfonic groups to the perfluorinated hydrophobic backbone, which results in the formation of a highly conductive medium for protons to provide greater reticulation of the nanocomposites on the surface of the GCE. Finally, the modified electrode (F_0.1_BiVO_4_/r-GO/Nafion) was utilized as an ECS to detect 4-CP. The detection limit of F_0.1_BiVO_4_/r-GO/Nafion towards 4-CP was low, showing that it has the advantages of the combination of composite materials, such as fast electron transport rate, large specific surface area and high conductance.

## Experimental

### Materials

In this work, bismuth (III) nitrate pentahydrate (Bi (NO)_3_⋅5H_2_O, 98.0%), was purchased from Fluka and ammonium vanadate (NH_4_VO_3_, 97.0%), ethylene glycol (EG) (MW: 20,000), sodium fluoride (NaF), and the graphite powder (< 50.0 mg) were purchased from Merck and they were used in the hydrothermal synthesis of F_0.1_BiVO_4_/r-GO/Nafion composite. Also, 4-chlorophenol was purchased from Across Company. We prepared the Britton–Robinson buffer solution (0.1M BR, pH 3.0–10.0) through mixing the standard stock solution of Boric acid (H_3_BO_3_) (99.8% ACS reagent, Sigma-Aldrich). Diluted Phosphoric acid (H_3_PO_4_) (97% ACS reagent, Sigma-Aldrich) and Acetic acid (CH_3_COOH) (99.5%, Alfa Assar) were used for the preparation of 3.0–10.0 pH during the study.

### Characterization

We recorded the FT-IR spectra on a thermoscientific NICOLET IR100 FT-IR Spectrometer which ranged from 400 to 4000 cm^−1^ using the KBr disk technique. A Renishaw instrument, model InVia reflex equipped with 532, and 785 nm lasers, was used to obtain the Raman spectra. The laser power was 10 mV. The spectral resolution was < 3 cm^−1^. For all spectra, acquisition time was 60 ms (4 × 15 ms). The Raman spectra of each sample were filed at diverse areas of the sample. The phase formation of samples was investigated by performing PXRD through a X'Pert diffractometer of PAN analytical with monochromated Co-Kα (λ = 1.78901 Å) radiation, in the 2θ range of 5° to 90°. The characterization of the electrode’s surface structure was investigated through a scanning electron (FE-SEM, Te-Scan-Mira, Czechia, Europe) microscope together with an Energy-dispersive X-ray spectrometer (EDX, X-Max 50, Czechia, Europe). UV–Vis diffuse reflectance spectrometer (Agilent, USA, BaSO_4_ is the reference) was employed for testing the UV–Vis absorption spectra of the composite. The electroanalytical experiments were performed through an EG&G PARSTAT 2273 electrochemical potentiated with a conventional three-electrode unit. The F_0.1_BiVO_4_/r-GO/Nafion-coated glassy carbon electrode (GCE) was the working electrode, and an Ag/AgCl electrode in aqueous 3.0 M KCl solution and Pt, respectively, were utilized as reference and counter electrode. The DPV scan was performed in a potential range of 0.0 to 1.8 V with a step potential of 8.5 mV, a pulse amplitude of 10 mV, a pulse width of 10 ms, a pulse period of 100 ms, and a scan rate of 0.01–0.07 V/s. The scan rate was calculated as the potential increment per unit time during the DPV scan.

### Preparation of P-BiVO_4_ and F_0.1_BiVO_4_

Spherical-like BiVO_4_ was synthesized using a hydrothermal reaction, according to the prior reports^28^. For the typical preparation, we dissolved 2.0 mmol of Bi (NO_3_)_3_·5H_2_O in 30.0 mL of ethylene glycol (EG) solution under continuous agitation at room temperature for 30 min. Next, we dissolved 0.234 g of NH_4_VO_3_ in 20.0 mL of EG for 15 min under steady stirring. We added NH_4_VO_3_ to the above mixture dropwise and a homogeneous solution was established following 1 h stirring. Then, we added NaF with the molar ratio of 0.5 wt% to the above solution and it was stirred for another hour. We kept the obtained solution in a Teflon-lined autoclave for 24 h at 180 °C. Following the reaction, we later collected the black precipitate through centrifugation using water and ethanol was used to wash it several times. We dried the obtained powders in a vacuum oven for 20 h at 60 °C and after that they were calcined at 450 °C for 5 h.

### Fabrication of F_0.1_BiVO_4_/r-GO Composite

The synthesis of GO nanosheets was accomplished through the chemical oxidation of graphite flakes and then exfoliation, according to the improved Hummers’ method^29^. The standard hydrothermal method was adopted to fabricate F_0.1_BiVO_4_/r-GO composites. In summary, we dispersed 0.05 g of F-BiVO_4_ powder in 50.0 mL of deionized water and ethanol through ultra-sonication for 15 min. Afterward, we introduced 50.0 mL of GO suspension (2.5 mg/mL) into the F_0.1_BiVO_4_ suspension dropwise, obtaining the weight ratio of GO to F_0.1_BiVO_4_ at 0.0025:0.05. After stirring at 60 °C for 5 h, a homogeneous suspension was created. The F_0.1_BiVO_4_/r-GO suspension was then rinsed with ethanol and deionized water several times and dried in a 60 °C oven. The synthesis method’s schematic view is presented in Fig. 1.

### Fabrication of F_0.1_BiVO_4_/r-GO/Nafion

To prepare the working electrode, 2–10 mg of the prepared materials were dispersed in 1 mL of ethanol and isopropanol with a volume ratio of 1:1 and 15 µL of 0.1 wt% Nafion suspension under sonication for 30 min to form a homogeneous ink. Then, the bare GCE was polished with 0.3 μm Al_2_O_3_ slurry and ultrasonically cleaned in distilled water and ethanol several times. Then, 2.5 µL of the prepared ink was loaded onto a polished GCE and dried. Different modified-electrodes were characterized by cyclic voltammetry (CV) in 5 mM [Fe (CN)_6_]^3−/4−^ containing 0.1 M KCl.

## Results

### Material characterizations

**Figure 2:**
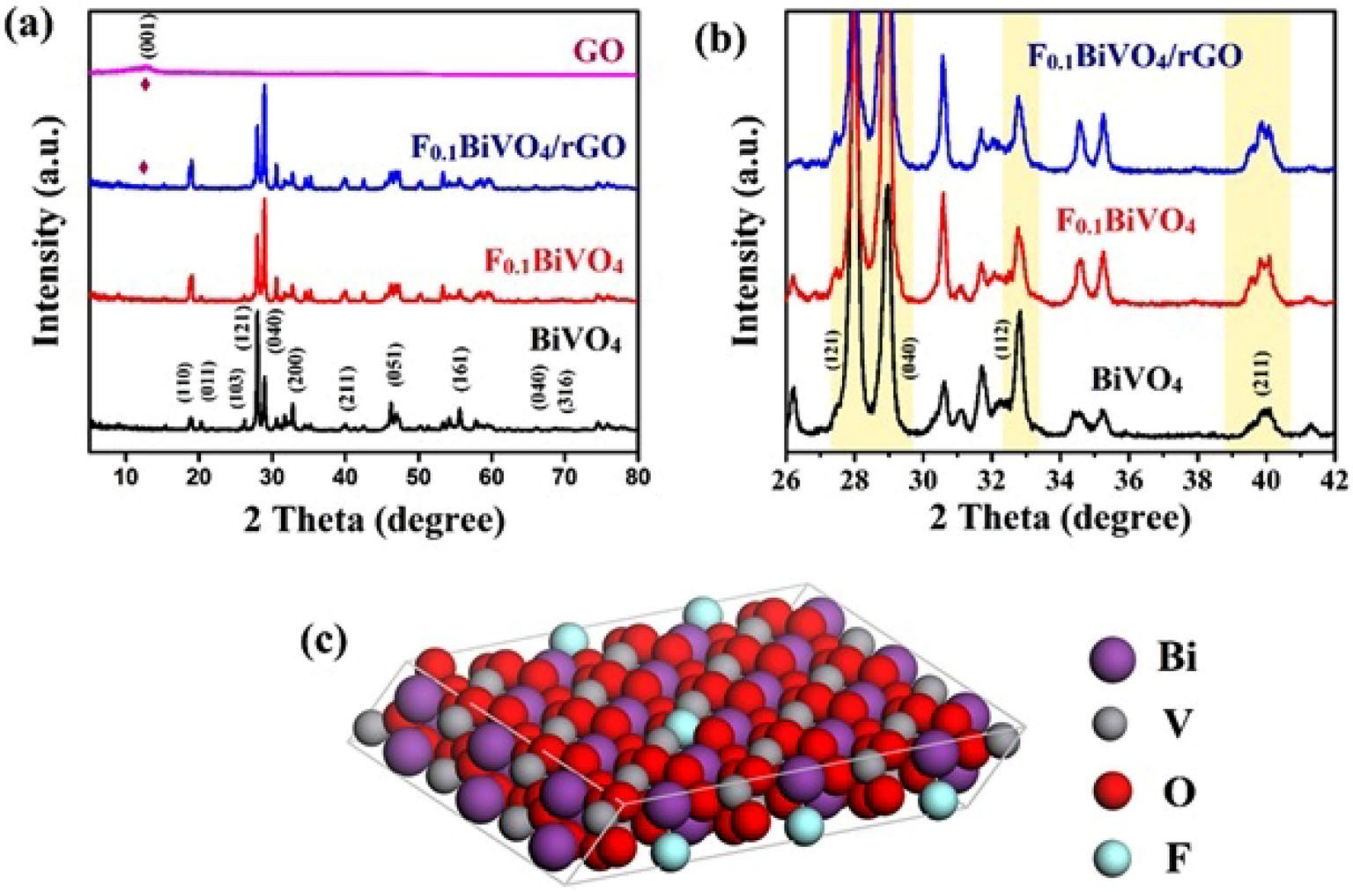
(**a**) XRD patterns of BiVO_4_, F_0.1_BiVO_4_, F_0.1_BiVO_4_/r-GO/Nafion and GO; (**b**) the magnified patterns; (**c**) schematic illustration of F_0.1_BiVO_4_ structure.

XRD in the 2θ range of 5° to 90° (see Fig. 2) was used to analyze the crystal phases of the BiVO_4_ and F-BiVO_4_ nanostructures. We can index the XRD patterns of P-BiVO_4_ to the characteristic peaks (CPs) of monoclinic BiVO_4_ (JCPDS 83-1697, m-BiVO_4_), where the peaks located at 19.0°, 30.0°,30.5°, 35.2°, 46.7°, 53.3°, and 59.5° belong to the (011), (112), (004), (020), (204), (116) and (132) planes, respectively. The pattern of F-BiVO_4_ exhibited the main peaks of m-BiVO_4_. Meanwhile, the CPs of the BiVO_4_ at 30.6°, 32.8°, and 39.9° shift to lower angles after modification with fluorine (see Fig. 2b). The observed expansion in the crystal lattice can be related to the well incorporation of F into the lattice of BiVO_4_, due to the fact F has a smaller ion radius (1.19 Å) than o2 (1.28 A)^30–32^. Also, the XRD pattern of the F_0.1_BiVO_4_ shows an increase in the peak intensity at 2θ = 27.5°, 30° compared with that of BiVO_4_, suggesting that F-doping leads to the prompt of the crystallization of V_2_O_5_. Using the Debye–Scherrer equation, we obtained the average size of the crystal particles, which is equal to 45.5 nm. Moreover, the dipole moment (DM) of VO_4_^3−^ tetrahedron deviated from zero to non-zero DM after BiVO_4_ was doped with F, which can increase the separation of charge on photo-excitation and improve the electrochemical activity^20,26,32,33^. The 4-CP of GO (001) at around 11° almost disappears in the r-GO-BiVO_4_ nanocomposite, which might be because of the low content of GO used in the nanocomposites^21,34,35^.

**Figure 3:**
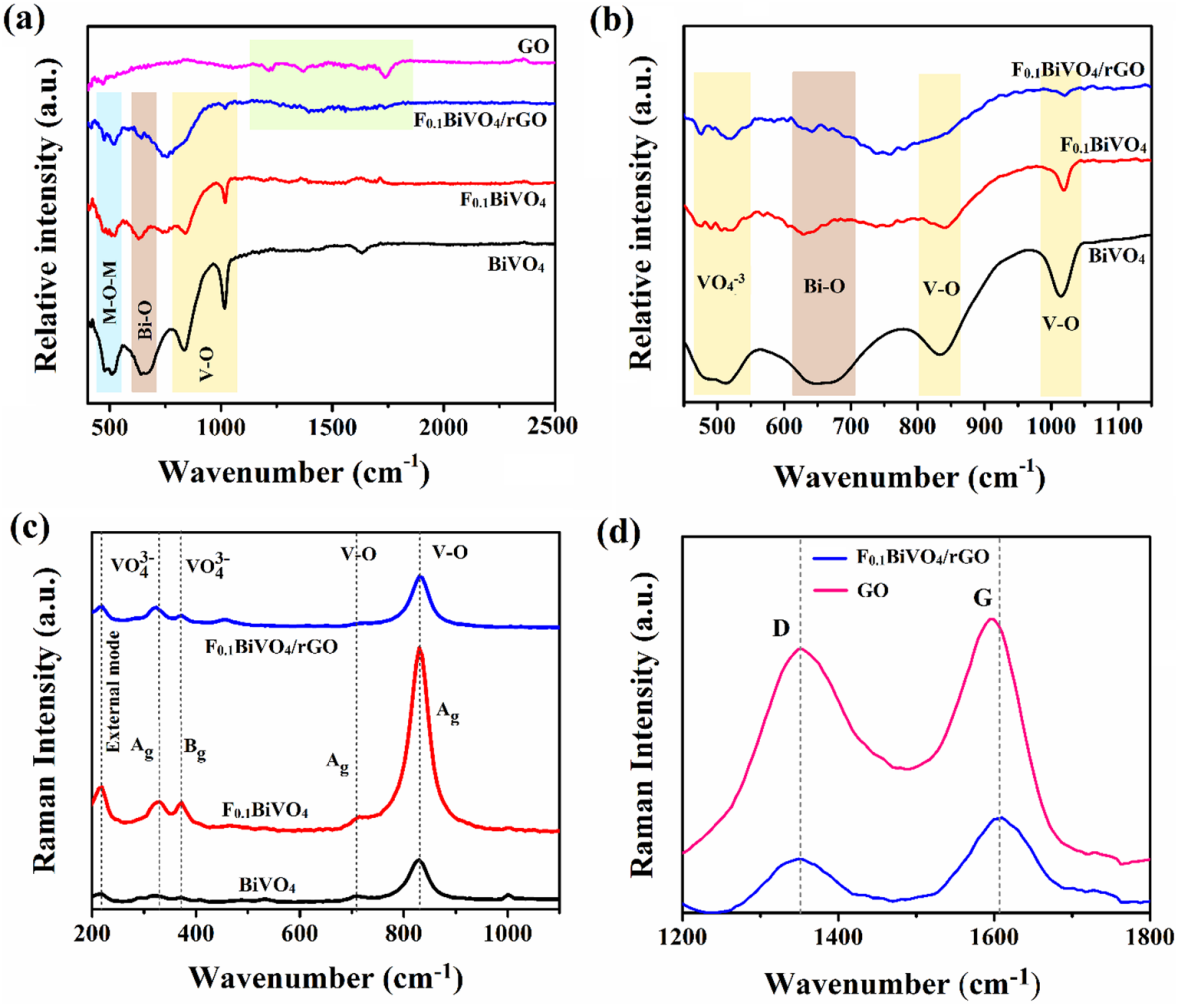
(**a**) FT-IR spectra of the as-synthesized samples; (**b**) The magnified FT-IR spectra; (**c**) Raman spectra and the magnified peaks of BiVO_4_, F_0.1_BiVO_4_, F_0.1_BiVO_4_/r-GO (**d**) Raman spectra and the magnified peaks of F_0.1_BiVO_4_/r-GO and bare r-GO.

We recorded the FTIR measurements of P-BiVO_4_, F-doped BiVO_4_, and F-doped BiVO_4_/r-GO/Nafion composite in the range of 400–4000 cm^−1^ and Fig. 3 demonstrates the related results. Figure 2a demonstrates the characteristic bands of the BiVO_4_ and F-doped BiVO_4_.The peaks around, 800 cm^−1^, and 1100 cm^−1^ were attributed to the stretching mode of VO_4_^–3^^36,37^, and its branch at 650 cm^−1^ was ascribed to Bi-O^17,38^, and also peak at 745 cm^−1^ was because of V–F vibration^20,33^. The presence of V–F vibration corroborated the insertion of F atoms into the crystal lattice of BiVO_4_. Figure 3b presents the FTIR spectra of GO and F_0.1_BiVO_4_/r-GO/Nafion overlapped for an exact comparison. The GO spectra demonstrated numerous vibrational peaks that were strong, corresponding to different O functional groups. Because of OH stretching vibration, a strong GO absorption band was observed at 3410 cm^−1^^39,40^. The CPs at 1725, 1371, 1211, and 1062 cm^−1^ could be attributed to carboxyl or carbonyl C=O stretching, carboxyl–OH stretching, C=C stretching, and alkoxy C–O stretching, respectively^23,41^. In the spectrum of the nanocomposite, F_0.1_BiVO_4_/r-GO/Nafion, there was a substantial reduction in the intensity of those peaks related to O functional groups, which revealed that GO was reduced into r-GO. The M–O–C bonds (M = V or Bi), usually appearing below 900 cm^−1^, were observed around 810 cm^−1^ and demonstrated that metal oxide interacted with Gr^22,24^.

**Figure 4:**
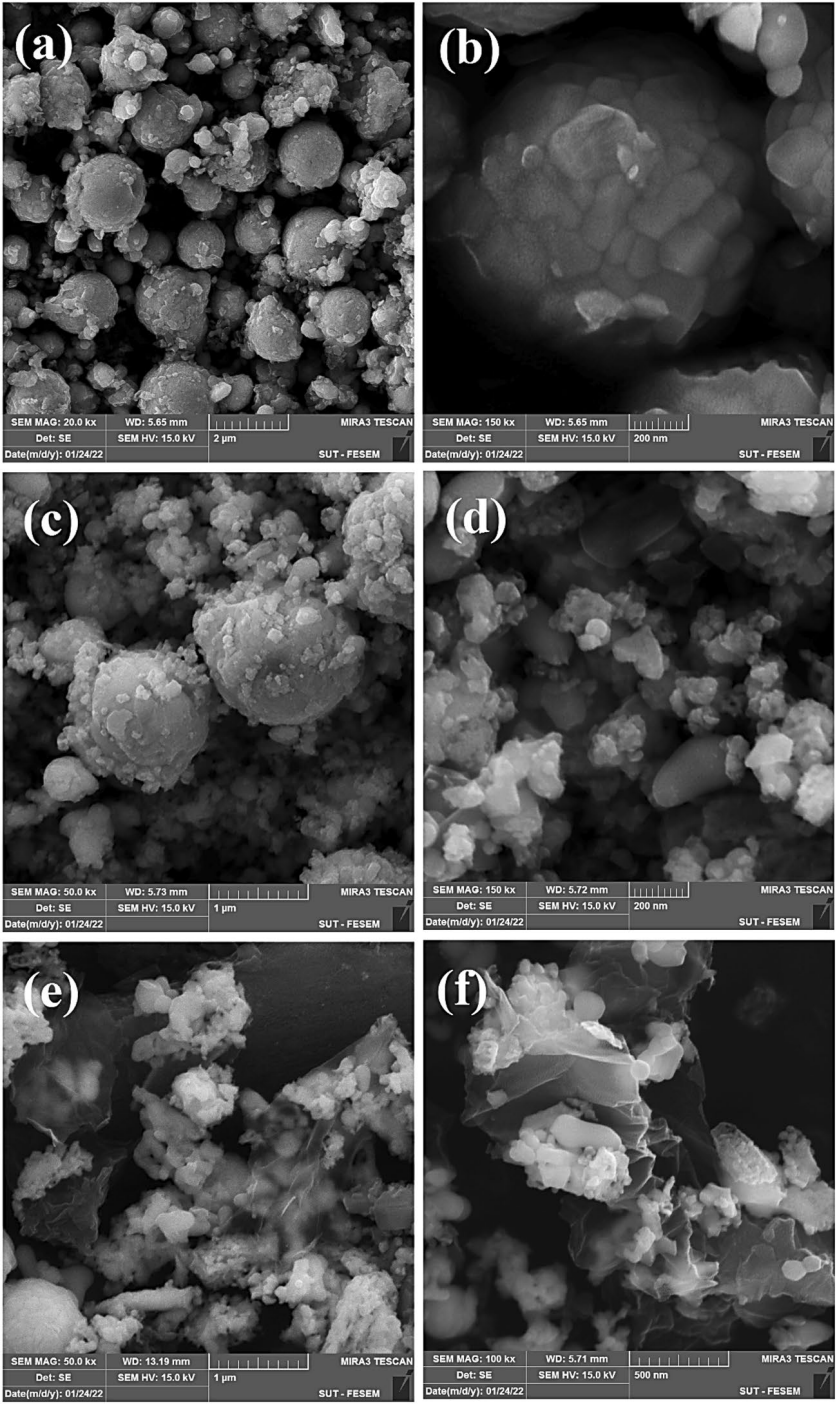
FE-SEM images of (**a**,**b**) BiVO_4_; (**c**,**d**) F_0.1_BiVO_4_ and (**e**,**f**) F_0.1_BiVO_4_/r-GO nanocomposite.

An efficient method for inspecting the crystallization, electronic properties of the materials and investigating the local structures is Raman spectroscopy. Figure 4a demonstrates the Raman bands and the presence of BiVO_4_ in all of the samples that were synthesized. It was observed that BiVO_4_ had a monoclinic phase according to the attributed stretching vibrations and twisting vibrations of the VO_4_^3–^ tetrahedron^18,42^. The strongest Raman band around 831 cm was assigned to the symmetric V–O (A_g_) stretching mode, but the weak Raman band at 706 cm was assigned to the antisymmetric V–O (B_g_) mode stretch^19,43^. The Raman band near 372 represents the symmetric δ_s_ (VO_4_^3–^) (A_g_) bending mode and 331 cm^−1^ represents the antisymmetric δ_as_ (VO_4_^3–^) (B_g_) bending mode.

The mode at 213.23 cm^−1^ was assigned as rotational and 126.58 cm^−1^ was assigned as translational. As shown in the inset of Fig. 3, the intensity of the bands increased in the Raman peak of F-doped BiVO_4_ indicates (defect formation) that the VO_4_^3−^ tetrahedron was weakly deformed, which was due to the fact that V ions were replaced with F ions in the F-doped sample, we could not observe extra peaks, but there was an enhancement in the intensity of the peak for F-doping, which was incorporated in the BiVO_4_ and also increased the crystallinity behavior observed in XRD data. On the other hand, the Raman profile of GO was dominated by 2 CPs of carbonaceous materials located at 1351 and 1590 cm^−1^, i.e., the D band peak because of the sp^3^ defects and the G band peak, which could be attributed to in-plane vibrations of sp^2^ C atoms^44,45^. The Raman spectrum of F-doped BiVO_4_/r-GO composite exhibited the same CPs as F doping in BiVO_4_ and G and D bands of GO. Additionally, peak G in F_0.1_BiVO_4_/r-GO was relatively blue-shifted by 20 cm^−1^ in contrast with GO. Therefore, this clearly demonstrated that GO was effectively reduced into r-GO. The intensity ratios of the D and G bands (I_D_/I_G_) in GO and F_0.1_BiVO_4_/r-GO were determined 0.93 and 0.95, respectively. The I_D_/I_G_ ratios for F_0.1_BiVO_4_/r-GO was higher than those for GO, indicating that a significant number of structural defects were introduced to the graphene lattice in the reaction. Conclusively, the larger I_D_/I_G_ ratios in the F_0.1_BiVO_4_/r-GO compared to GO indicate an increase in the amount of smaller sp^2^ domains and the revival of graphene network conjugation (re-aromatization). Moreover, the revived graphene network size was smaller than that of the GO starting material. This effect gave rise to an increased I_D_/I_G_ ratio in the F_0.1_BiVO_4_/r-GO composite materials.

**Figure 5:**
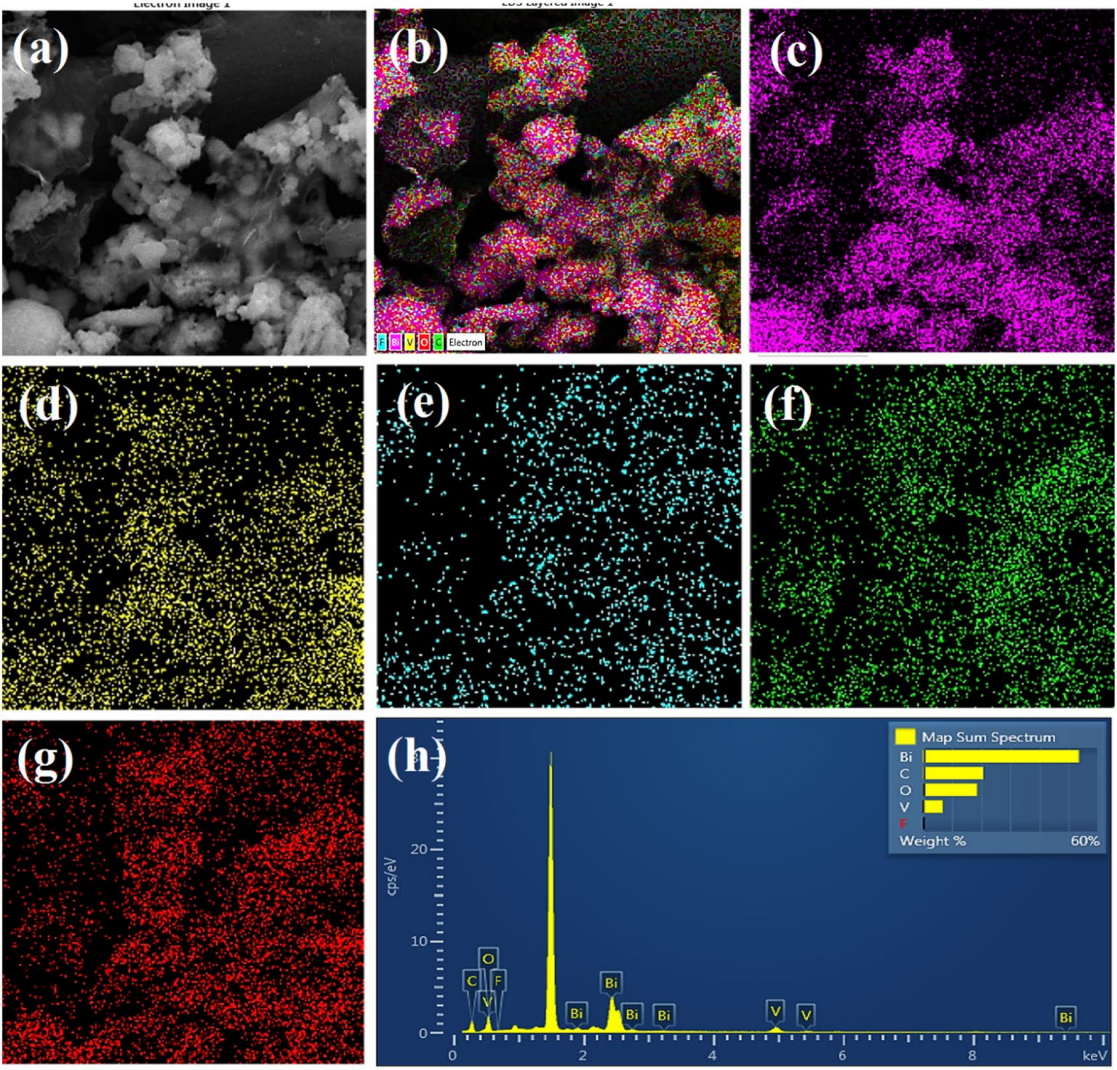
(**a**–**g**) EDS-mapping images of F_0.1_BiVO_4_/r-GO nanocomposite; (**h**) The corresponding EDS spectrum.

FE-SEM micrographs (see Fig. 4) show images of un-doped and F-doped BiVO_4_, and F_0.1_BiVO_4_/r-GO samples. Figure 4a shows that the structure of BiVO_4_ has spherical-like and homogenous particles with diameters of 40–90 nm. The magnified images in Fig. 4b indicate that the spherical particles are formed by agglomeration of smaller polyhedral grain-like nanoparticles with size of 15 nm. Figure 4c and d show the SEM images of the F-BV sample which illustrates smaller nanoparticles than the P-BiVO_4_. After the composition of F_0.1_BiVO_4_ with GO (Fig. 4e and f), the F_0.1_BiVO_4_ nano-particles had a uniform distribution on the GO surface. The good distribution of nanoparticles on GO nanosheets could provide more active sites, which is beneficial for enhanced electrochemical performance. Additionally, the elemental distribution of C, O, Bi, and F in F_0.1_BiVO_4_/r-GO nanocomposite was achieved through energy dispersive spectroscopy (EDS) analysis. The results from mapping images in Fig. 5 and Table 1S confirmed that all of the essential elements were present and Fluorine atoms were distributed well in the BiVO_4_ structure.

### Fabrication of modified electrodes

To prepare the working electrode, 2–10 mg of the F_0.1_BiVO_4_/r-GO was dispersed in 1 mL of ethanol and isopropanol with a volume ratio of 1:1 and 15 µL of 0.1 wt% Nafion suspension under sonication for 30 min to form a homogeneous ink. Then, the bare GCE was polished with 0.3 μm Al_2_O_3_ slurry and ultrasonically cleaned in distilled water and ethanol several times. Then, 2.5 µL of the prepared ink was loaded onto a polished GCE and dried. For comparison, a similar procedure was used to prepare F_0.1_BiVO4/GCE, GO/GCE, and BiVO_4_/GCE. Different modified-electrodes were characterized by cyclic voltammetry (CV) in "5 mM" [Fe (CN)_6_]^3−/4−^ containing 0.1 M KCl.

### Electrochemical characterization

**Figure 6:**
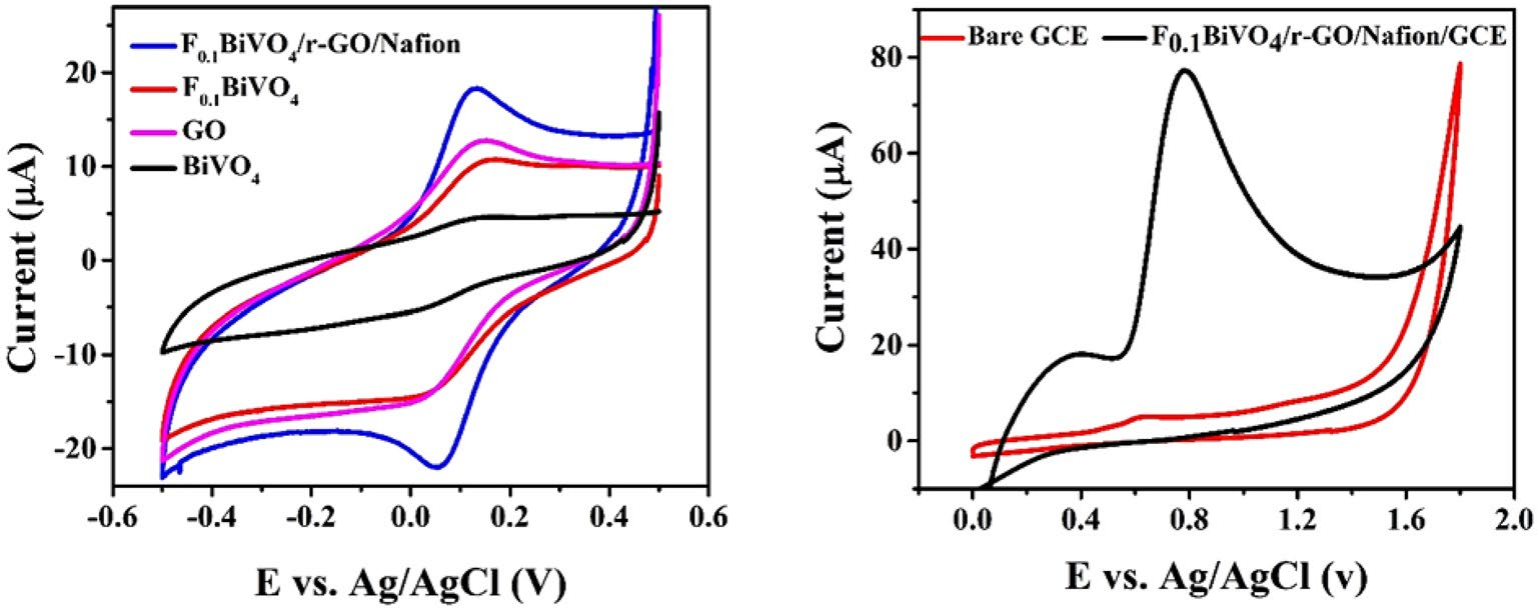
(**a**) Cyclic voltammogram of BiVO_4_/GCE, F_0.1_BiVO_4_/GCE and F_0.1_BiVO_4_/r-GO/Nafion/GCE in the solution of 3 mM [Fe(CN)6]^3−/4−^ containing 0.1 M KCl with the potienal range − 0.5 to 0.5 V vs. Ag/AgCl; (**b**) Electrochemical behavior of F_0.1_BiVO_4_/r-GO/Nafion /GCE and bare GCE at scan rate 0.1 Vs^−1^ in presence 1 nM 4-CP in 0.1 M BRB (pH∼7.0).

The electrochemical behavior of (black) BiVO_4_/GCE, (red) F_0.1_BiVO_4_/GCE, (Pink) GO/GCE, (Blue) F_0.1_BiVO_4_/r-GO/Nafion/GCE was thoroughly inspected through the measurement of the CV in solution of [Fe (CN)_6_]^3−/4−^ with 0.1 M KCl with the frequencies at 50.0 mV/s scan rate. As shown, the values of anodic peak current of these electrodes were as follows: 2.46 μA (Bivo4/GCE), 7.9 μA (F_0.1_BiVO_4_/r-GO/Nafion/GCE), 8.8 μA (GO/GCE), and 16.5 μA (F_0.1_BiVO_4_/r-GO/Nafion/GCE). Using the CV results, it was concluded that the F_0.1_BiVO_4_/r-GO/Nafion on the electrodes surface exhibited the highest redox peak currents. This was attributed to the large surface area and the excellent electrical conductivity of the GO and F_0.1_BiVO_4_, which significantly increased the electron transfer rate (see Fig. 6a)^46,47^. It was deduced from the extrapolated ΔE values that F_0.1_BiVO_4_/r-GO/Nafion (0.073 V) had a better electron transfer, followed by F_0.1_BiVO_4_ (0.095 V), BiVO_4_ (0.098 V), and GO (0.110 V). The indication is that F_0.1_BiVO_4_/r-GO/Nafion is surpassing GO in terms of conductivity^46,47^.

Electrochemical behavior of F_0.1_BiVO_4_/r-GO/Nafion/GCE,and bare GCE electrodes in the presence of 0.1 nM of 4-CP (in 0.1 M KCl at pH∼7.0) were scrutinized through cyclic voltammetry at 0.1V/s scan rate. In the in the presence of GCE electrode, no redox peak was observed. But in the presence of F_0.1_BiVO_4_/r-GO/Nafion/GCE, a relatively large oxidation peak with the potential of 0.78 V and the peak current of about 58 μA was observed (see Fig. 6b), revealing that F_0.1_BiVO_4_/r-GO/Nafion/GCE served as an efficient electron promoter for the 4-CP electrocatalytic oxidation. (Also, different percentages of fluorine dopant atoms were investigated. This is visible in Fig. 2S where the sample with a 10% dopant concentration showed the highest current among the other samples and was chosen as the optimal sample for further investigation).

**Figure 7:**
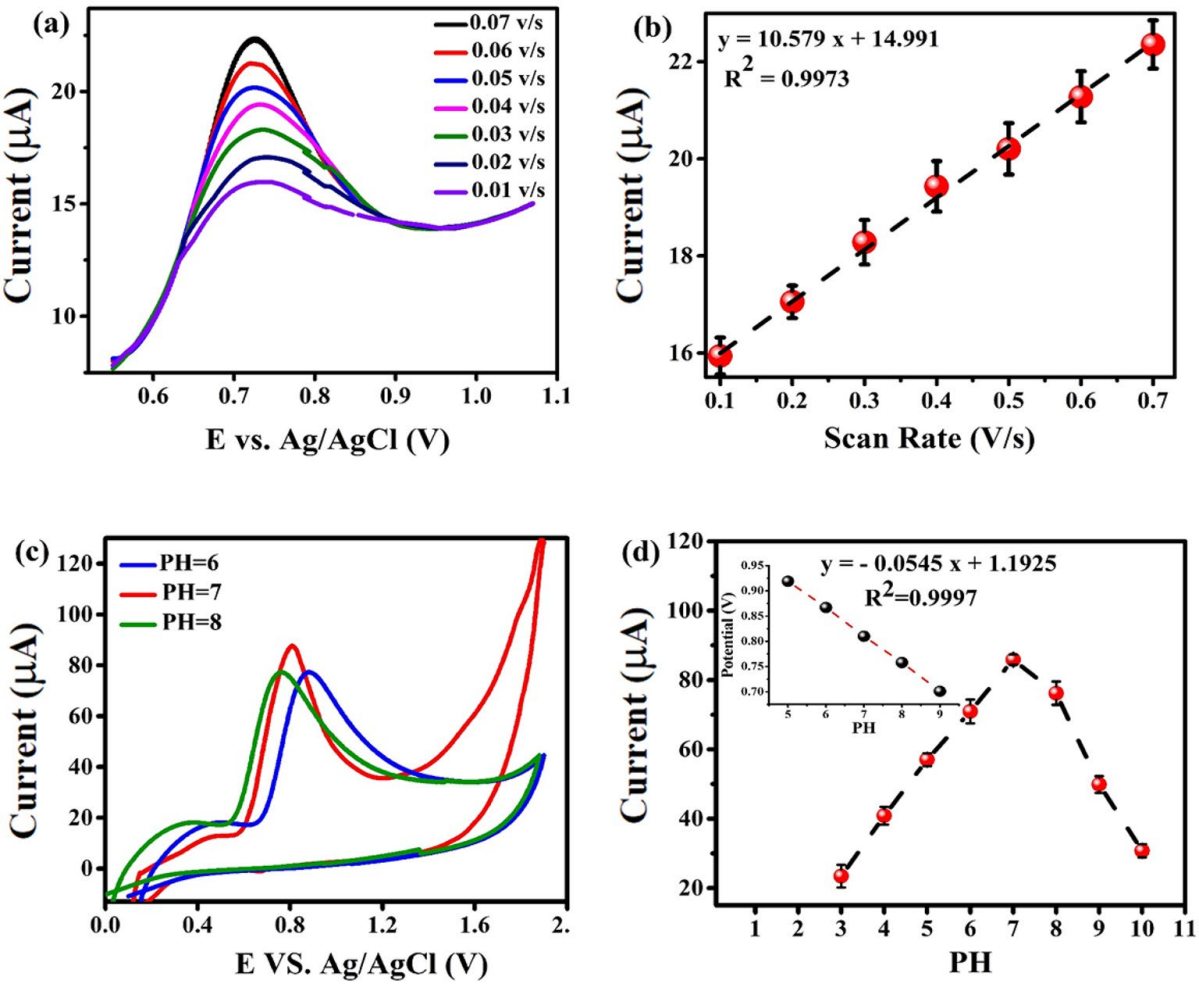
(**a**) Differential pulse voltammetry curves for F_0.1_BiVO_4_ /r-GO/Nafion/GCE in present of 1 nM 4-CP in 0.1 M KCl (pH 7.0) with different scan rates; (**b**) Linear relationship between the oxidation peak currents and the scan rates; (**c**) CVs of F_0.1_BiVO_4_ /r-GO/Nafion/GCE in present 1 nM 4-CP in 0.1M BRB, with different pH values: 3.0, 4.0, 5.0, 6.0, 7.0, 8.0. Scan rate 0.01 Vs^−1^; (**d**) Influences of pH on the oxidative peak current and oxidative peak potential.

The DPVs of 4-CP with varying potential scan rates (0.01 to 0.07 V/s) on F_0.1_BiVO_4_/r-GO/Nafion/GCE were investigated to further understand the sensing mechanism of 4-CP (see Fig. 7a). In the Figure, a phenomenon we observed was that the oxidation peak potential did not shift much, and the peak currents gradually increased with increasing scanning speed. As can be seen in Fig. 7a,b, while there was an increase in scan rate from 0.01 to 0.07 V/s, the oxidation peaks current linearly increased. There was a linear dependence between the scan rate and the anodic peak current of 4-CP, and it obeys the following equation:

$${\text{Ip}}_{{\text{a}}} \left( {\upmu {\text{A}}} \right) = 10.597\upnu \left( {{\text{mV S}}^{{ - 1}} } \right) + 14.991\left( {{\text{R}}^{2} = 0.9973} \right)$$

The results show that the electrochemical response process of 4-CP onto the F_0.1_BiVO_4_/r-GO/Nafion/GCE sensor surface is controlled by adsorption.

The impact of pH on the CV responses of F_0.1_BiVO_4_/r-GO/Nafion/GCE was evaluated in 0.1 M Britton–Robinson buffer (BRB) in the pH range of 3.0 to 10.0 (see Figs. 7c,d, and 1S). Hence, pH = 7.0 is assigned as the standard pH for detecting 4-CP in this work. However, when the pH values are enhanced from 8.0 to 10.0, a decrease in the peak current occurs, which might be because of the electrostatic repulsion of anionic 4-CP which had negative charges on the surface of the sensor. Furthermore, the oxidation (Ep_a_) peak potential became lower and lower by increasing pH. Also, we can express the dependence of pH on the shift in Ep_a_ as follows^48,49^.

$${\text{Ep}}_{{\text{a}}} (\text{v}) = -0.0545 \times \mathrm{ PH }+ 1.997 ({\text{R}}^{2}=0.999)$$

**Figure 8:**
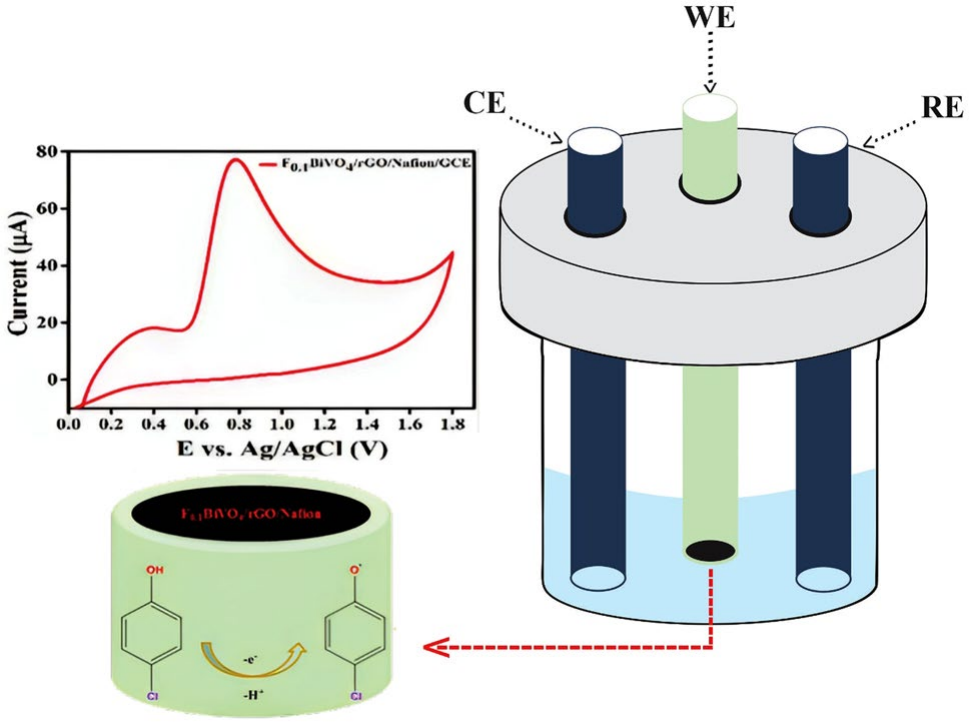
The electron transfer mechanism and electrochemical reaction performance in 4-chlorophenol on the GCE modified with F_0.1_BiVO_4_/r-GO/Nafion.

This slope value is approximately the same as the theoretical Nernstian number of − 59.0 mV/pH, which demonstrated that the number of involved protons and electrons in the electron transfer process of 4-CP was equal. Therefore, it can be inferred that the electrochemical oxidation of 4-CP at the F_0.1_BiVO_4_/r-GO/Nafion/GCE electrode is a one-electron and one-proton process and this is illustrated in Fig. 8^50^.

**Figure 9:**
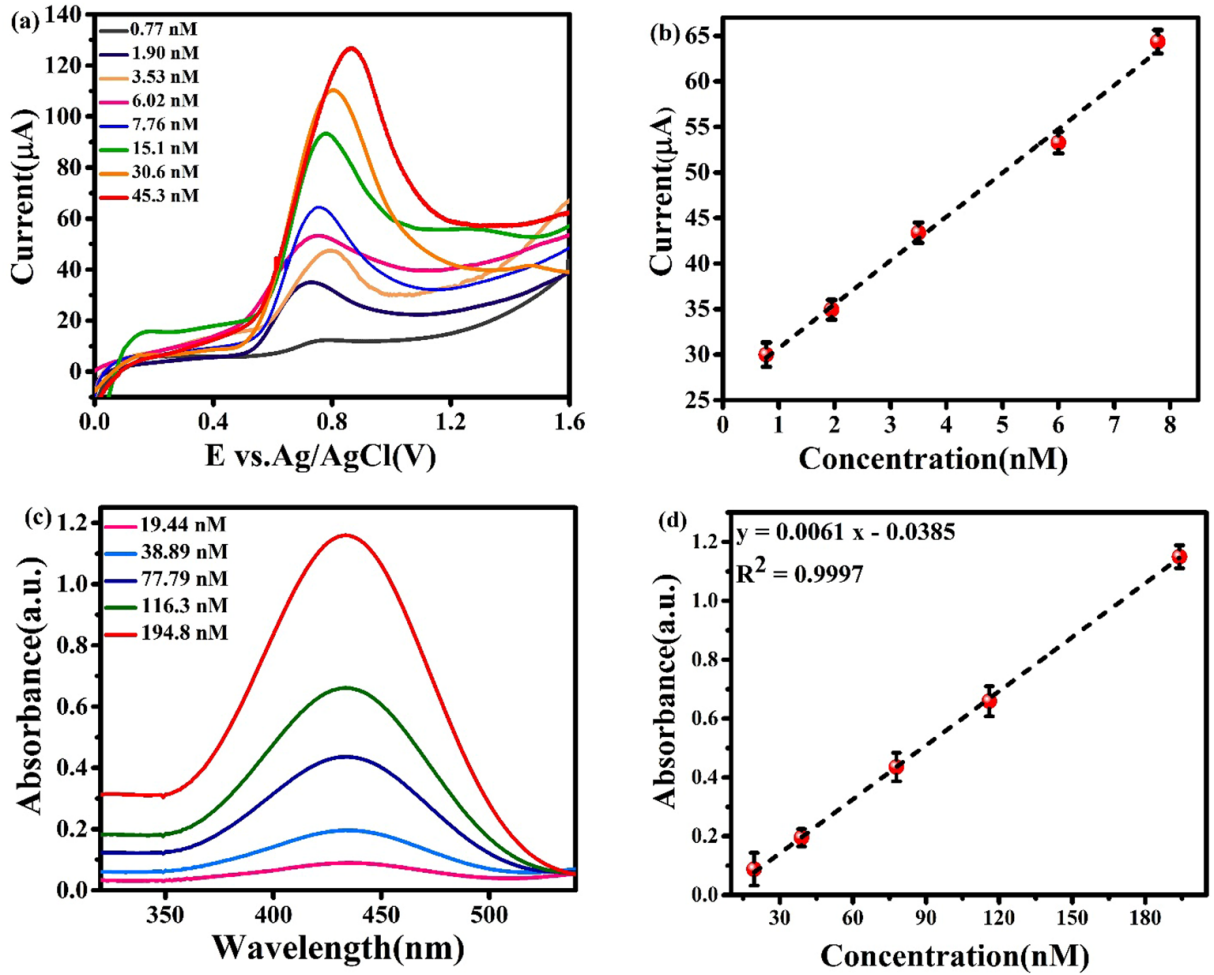
(**a**) Differential pulse voltammograms for 4-CP with various concentrations; (**b**) Calibration plots of the oxidation current at F_0.1_BiVO_4_/r-GO/Nafion/GCE versus concentration of 4-CP under optimal conditions, (**c**) Uv–Vis spectra for 4-CP with various concentrations and (**d**) Calibration plots of F_0.1_BiVO_4_/r-GO/Nafion/GCE form Uv–Vis analysis.

Under the optimal conditions, the analytical ability of the F_0.1_BiVO_4_/r-GO/Nafion/GCE sensor for detecting a series of 4-CP solutions with varying concentrations was investigated by the DPV method. As exhibited in Fig. 9a, a linear increase was observed in the oxidation peaks current of 4-CP (I_p_) by increasing the concentration of 4-CP from 0.77 to 45.0 nM. As can be seen from Fig. 9b, we found that concentration and the oxidation peak current are closely related:

$${\text{Ip}}_{{\text{a}}} (\upmu\text{A}) = 4.8126\mathrm{ c }+ 25.942(\upmu \mathrm{M}) ({\text{R}}^{2}=0.9949)$$

The detection limit for 4-CP was 0.56 nM (S/N = 3). Moreover, in Table 1, we compared the LOD values and the linear range with other previous reference for detecting 4-CP with electrodes based on GO. The data showed that the linear range was wider; the detection limit was lower, the sensitiveness was higher, the specific surface area was larger, and the electrocatalytic activity was higher for the 4-CP which was developed using the nanocomposite structure F_0.1_BiVO_4_ and GO thin nanosheets, which were better than those of previously reported sensors.

**Table 1:** LOD comparison of F_0.1_BiVO_4_/r-GO/Nafion with other sensors reported on chlorophenol based martial.

Modified electrodes	Analyte	LOD	References
Ag@Nd_2_O_3_	4-Nitrophenol	0.43 pM	^51^
α-Fe_2_O_3_	Phenolic	2.0 nM	^52^
ZnMn2O4	Nitrophenol	20.0 mM	^53^
Er_2_O_3_/CuO	3-chlorophenol	0.083–0.002 nM	^54^
PANI@G/CWO	2-nitrophenol	0.87 nM	^55^
TMO	Bisphenol-A	1.2–0.1 nM	^56^
rGO/HAp	Bis-phenol A	60.0 pmolL^1^	^57^
Ce-doped ZnO	phenolic	11.5 0.2 pM	^58^
Cu-Au/r-GO	4-chlorophenol	0.10 × 10^−3^ nM	^59^
Cu/r-GO	4-chlorophenol	0.23 × 10^−3^ nM	^59^
Au/r-GO	4-chlorophenol	0.17 × 10^−3^ nM	^59^
GO-Fe_3_O_4_-G_4_ PAMAM/ILCPE	4-chlorophenol	20 nM	^60^
GO@LaVO_4_-NCs	4-chlorophenol	2.44 nM	^61^
CR-GO/GC-E	4-chlorophenol	19.36 × 10^3^ nM	^62^
F_0.1_BiVO_4_/r-GO/Nafion	4-chlorophenol	0.56 nM	This work

**Figure 10:**
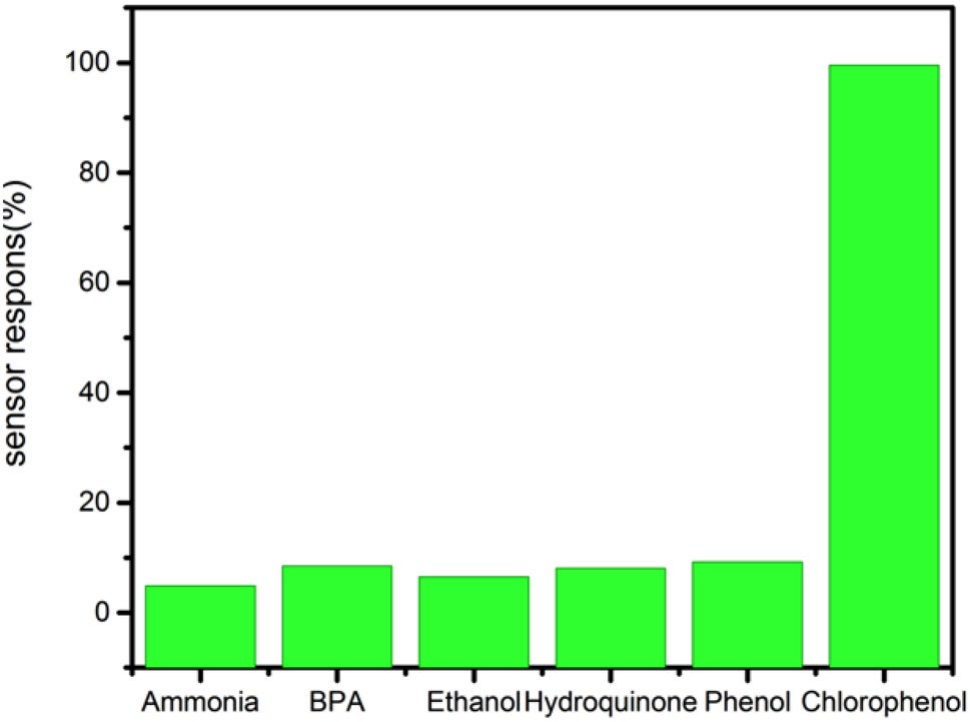
Influence of coexisting substances on the current response to different material by F_0.1_BiVO_4_/r-GO/Nafion/GCE in 0.1 M BRB (pH 7.0) containing 1 nM 4-CP and several interferences (Ammonia, BPA, Ethanol, hydroquinone, and phenol).

The selectivity of the F_0.1_BiVO_4_/r-GO/Nafion/GCE sensor was evaluated by studying the influence of some potential interferents including inorganic ions and organic phenolic compounds, which were coexisted in the electrochemical detection of 4-CP in the subsequent DPV experiments. As shown in Fig. 8, 10-fold higher concentrations of ammonia, BPA, ethanol, hydroquinone, and phenol did not affect the detection of 4-CP. As shown in Fig. 10, the sensitivity of the proposed F_0.1_BiVO_4_/r-GO/Nafion/GCE towards 4-CP was approximately 10 times larger than interferents, indicating the proposed sensor had good anti-interference ability, and therefore, can be used to selectively determine 4-CP.

**Figure 11:**
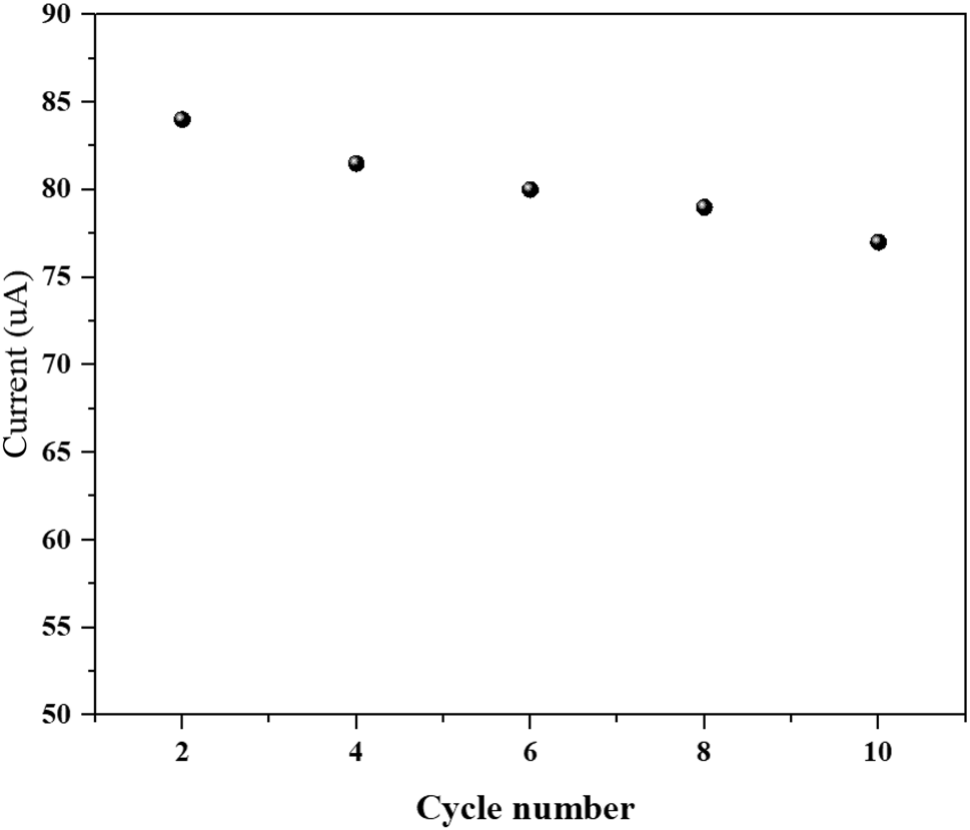
The repeatability of F_0.1_BiVO_4_/r-GO/Nafion/GCE in 0.1 M KCl (pH 7.0) containing 1 nM 4-CP.

Additionally, The oxidation peak current of 1 nM 4-CP decreased with the successive potential scan on the F_0.1_BiVO_4_/r-GO/Nafion/GCE in BR buffer solution (Figs. 11 and 3S).This situation can be due to the adsorbed 4-CP oxidation product and the polymerization of 4-CP on the electrode surface (which blocks electrode surface and obstructs further oxidation of 4-CP) which is in accordance with previous studies.As can be seen, the oxidation peak current reduced by 13% after 12 cycles. According to result, the F_0.1_BiVO_4_/r-GO/Nafion/GCE modified electrode effectively prevent the surface fouling effect caused by the oxidation products of the 4-CP. According to the result above, the developed F_0.1_BiVO_4_/r-GO/Nafion/GCE modified GCE is a suitable platform for the 4-CP analysis with good stability and repeatability.

To study the practicability of F_0.1_BiVO_4_/r-GO/Nafion/GCE modified electrode, real sample analysis was carried out using DPV technique. The standard addition method was followed to determine the 4-CP in tap water. The concentration of added PCMC and the recovery values are displayed in Table 2. The recoveries in the range of 101.10% to 102.73% were obtained, which suggested that the modified electrode might be applied for real sample tests.

**Table 2:** The recoveries of the prepared electrochemical sensor.

Samples	Current (μA)	RSD (%)	Recovery (%)
1	77.164	–	–
2	78.057	0.4465	101.15
3	78.921	0.7169	101.10
4	80.557	1.2516	102.07
5	82.757	1.9799	102.73

## Conclusions

In this study, the solvothermal method is applied to synthesize the catalyst F_0.1_BiVO_4_. In addition, an electrochemical method was applied to synthesize the F_0.1_BiVO_4_/r-GO/Nafion/GCE composite. Various techniques have been used to characterize the synthesized mixture for its morphological, structural and elemental properties. In addition, the 2D layered material synthesized with impregnated nanoparticles was exploited as an electrocatalyst for 4-CP detection. In addition, the quantitative determination of 4-CP through the proposed modified electrode provides a detection limit of 0.56 nM and a linear range of 0.77–45.0 nM, a wide range. Thus, the modified GCE demonstrated high sensitivity, better stability, and a lower detection limit for 4-CP detection. These properties demonstrated the potential practical applications of the F_0.1_BiVO_4_/r-GO/Nafion/GCE for 4-CP sensors. Furthermore, the new sensor is efficient, environmentally sustainable, cost-effective and simple to manufacture.

### Supplementary Information

Supplementary Information.

## Data Availability

The datasets generated and/or analyzed during the current study are available from the corresponding author upon reasonable request.
