# Supplementary material for: Development and application of fluorine doped bismuth vanadate reduced graphene oxide Nafion composite electrode as an electrochemical sensor for 4-chlorophenol
Source: Sci Rep. 2023 Dec 11;13:21912. doi: 10.1038/s41598-023-49205-y (PMC10713623; doi:10.1038/s41598-023-49205-y)
Supplement: Supplementary file 1 — Supplementary Information. [file 41598_2023_49205_MOESM1_ESM.docx]

**Supplementary information**

**Development and application of fluorine doped bismuth vanadate reduced graphene oxide nafion composite electrode as an electrochemical sensor for 4-chlorophenol**

Bahareh Ghorbannejad^a^, Alireza Mahjoub^a^* and Nima Dalir^b^

^a^Department of Inorganic Chemistry, Faculty of Basic Sciences, Tarbiat Modares University, P.O. Box 14115-175 Tehran, Iran.

^b^Department of Renewable Energy, Faculty of Interdisciplinary Science and Technologies, Tarbiat Modares University, P.O. Box 14115-175 Tehran, Iran

^*^Email address: Mahjouba@modares.ac.ir

s



**Fig. 1S:** Influences of pH on the CV responses of 1 nM 4CP at a scan rate of 0.01 Vs^-1^ in 0.1M BRB

**
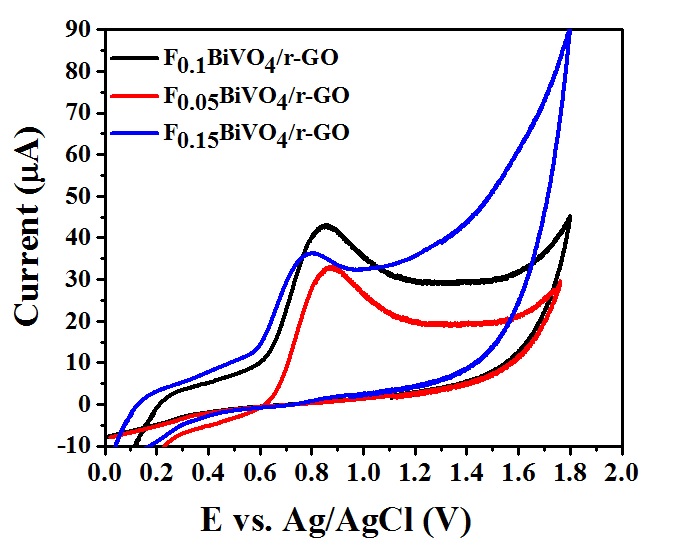
**

**Fig. 2S:** Influences of fluoride doped ratio on the CV responses of 1 nM 4CP at a scan rate of 0.01 Vs^-1^ in 0.1M BRB





**Fig. 3S**: The repeatability and stability of F_0.1_BiVO_4_/r-GO/GCE in 0.1 M BRB (pH 7.0) containing 1 nM 4CP.

**Table 1S**: Elemental mapping of O, V, Bi, F.

| Element | Wt% | Atomic % |
| --- | --- | --- |
| C | 20.67 | 52.25 |
| O | 18.58 | 35.28 |
| F | 0.38 | 0.60 |
| V | 6.87 | 4.09 |
| Bi | 53.51 | 7.78 |
| Total: | 100.00 | 100.00 |
